# Perspective on the Road toward Gene Therapy for Parkinson’s Disease

**DOI:** 10.3389/fnana.2016.00128

**Published:** 2017-01-09

**Authors:** Bas Blits, Harald Petry

**Affiliations:** Neurobiology Research, uniQure BVAmsterdam, Netherlands

**Keywords:** Parkinson’s disease, gene therapy, clinical trial, viral vector, dopamine, review, AAV, lentiviral vector

## Abstract

Many therapeutic strategies aimed at relieving symptoms of Parkinson’s disease (PD) are currently used for treatment of this disease. With a hallmark of progressive degeneration of dopaminergic neurons, the absence of properly operational dopaminergic circuitry becomes a therapeutic target. Following diagnosis, dopamine replacement can be given in the form of L-DOPA (L-3,4-dihydroxyphenylalanine). Even though it is recognized as standard of care, this treatment strategy does not prevent the affected neurons from degenerating. Therefore, studies have been performed using gene therapy (GT) to make dopamine (DA) available from within the brain using an artificial DA circuitry. One approach is to administer a GT aimed at delivering the key enzymes for DA synthesis using a lentiviral vector system (Palfi et al., 2014). A similar approach has been investigated with adeno-associated virus (AAV) expressing aromatic L-amino acid decarboxylase, tyrosine hydroxylase, and GTP-cyclohydrolase I (Bankiewicz et al., 2000), which are downregulated in PD. Another GT approach to mitigate symptoms of PD used AAV-mediated delivery of GAD-67 (glutamate decarboxylase) (Kaplitt et al., 2007). This approach mimics the inhibitory effect of DA neurons on their targets, in reducing motor abnormalities. Finally, disease modifying strategies have been undertaken using neurotrophic factors such as neurturin (NTN) (Marks et al., 2008; Bartus et al., 2013a) or are ongoing with the closely related Glial cell line-derived neurotrophic factor. Those approaches are aiming at rescuing the degenerating neurons. All of the above mentioned strategies have their own merits, but also some disadvantages. So far, none of clinical applied GT studies has resulted in significant clinical benefit, although some clinical studies are ongoing and results are expected over the next few years.

## Introduction

Gene therapy has shown encouraging results in preclinical models for numerous indications, which has led to a proliferation of clinical trials. For instance in the eye, where mutations in the *RPE65* gene are the cause of inherited retinal dystrophy, gene therapy was used to introduce the *RPE65* gene (Bennett et al., 2016), leading to a reversal in blindness in patients. In addition to the eye, the brain is an attractive organ for gene therapy and clinical trials in the central nervous system (CNS) are also emerging. With regards to delivery for a gene therapeutic approach, there are generally two options, local administration or systemic administration though intravenous injection or cerebrospinal fluid (CSF). It is well recognized that efficient DNA delivery is one of the critical obstacles in CNS gene therapy to eventually obtaining clinical benefit. The blood brain barrier (BBB) shields the brain from systemic delivery, and gene therapy products have not proven to efficiently cross the BBB in several animal models, with the exclusion of AAV-9 that is able to cross the BBB to a certain extent (Foust et al., 2008, 2009). Even though this AAV-9-mediated peripheral approach is very promising for the treatment of CNS diseases that affect the whole CNS, the transduction pattern following intravenous delivery to the brain does not specifically transduce the target structures for Parkinson’s disease (PD). Local delivery of a therapeutic gene may result in meaningful clinical results and has been the preferred option in clinical studies for PD.

**FIGURE 1:**
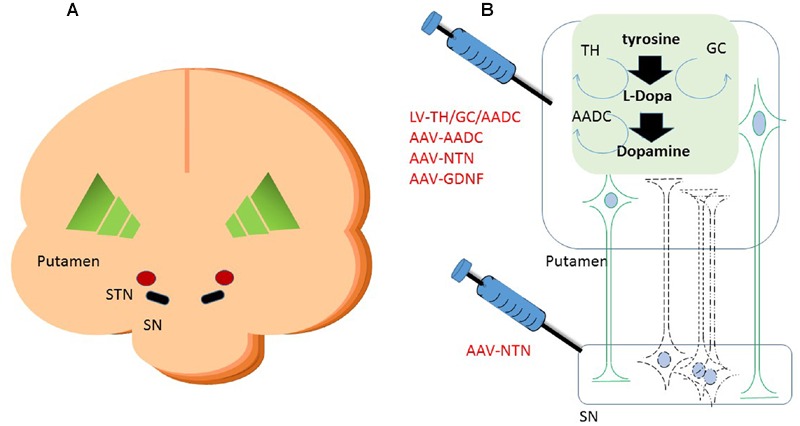
**Schematic cartoon of the brain with the schematic locations of the brain targets in a cross coronal plane **(A)**.** A schematic representation of gene therapeutic approaches to the putamen and the substantia nigra is shown in **(B)**. Neurons in black represent the DA neurons in the SN. The dashed lines are illustrating the degeneration of these neurons. In green are neurons depicted that project from putamen to SN. Upon putaminal transduction, neurons that project to the SN are able to anterogradely transport therapeutic proteins to the SN. For secreted proteins, such as neurotrophic factors, this results in depositing the possibly therapeutic protein to the targeted SN, irrespective of the status of the degenerating SN neurons. Retrograde transduction of the SN neurons via the putamen is also possible, but dependent on the status of the DA neurons. Therefore, injections have also been performed into the SN directly on top of intraputaminal injections. Moreover, in the putamen, some of the rate limiting enzymes of dopamine synthesis can be delivered via a gene therapeutic approach. AADC as one of the downregulated proteins in Parkinson’s disease (PD) has been delivered to the putamen via an adeno-associated virus (AAV) vector. Via a lentiviral vector approach, the three enzymes that are needed in the cascade of D synthesis, TH, AADC, and GCH, have been delivered. With the delivery of these enzymes, turnover of levodopa to dopamine should be brought to more physiologically healthy levels. SN, substantia nigra; STN, subthalamic nucleus; TH, tyrosine hydroxylase; AADC, aromatic L-amino acid decarboxylase; TH, tyrosine hydroxylase; GCH, GTP-cyclohydrolase I.

The option to apply a targeted local delivery makes PD a good candidate to test advanced therapies like GT as the affected neurons are located in a well described, relatively small nucleus, the substantia nigra (SN). Besides the most prevalent affected hallmark SN structure, many brainstem, limbic, and midbrain neurons are also subject to degeneration. Still, the hallmark of PD is the loss of dopaminergic neurons of the SN, which leads to alterations in the activity of brain networks that control movement (Obeso et al., 2000; Braak et al., 2003; Wichmann and DeLong, 2003). With an uncertain etiology for dopaminergic loss, several gene therapeutic strategies have been undertaken that have led to clinical studies (for an overview, see **Table 1**). To reach the SN, direct injections into the location can be performed (Bartus et al., 2013a), or indirect targeting using neuroanatomical interpretation of known connections within the brain. For instance, neurons projecting from the putamen to or from the SN can be used to reach the SN (see **Figure 1**). Based on this assumption secreted molecules like GDNF or neurturin (NTN) can be delivered to the SN by transducing the putamen (Kells et al., 2012; Bartus et al., 2013b). This has additional merit as the nigrostriatal neurons are the neurons that degenerate, and, irrespectively of the disease state, the neurons that are projecting from the putamen to the SN remain intact and thus can be used (Kells et al., 2010; Ciesielska et al., 2011).

**Table 1:** Summary of gene therapy clinical studies programs for Parkinson’s disease.

Product	Highest dose (GC total)	Volume (μl)	Target	Mode of action	Sponsor	Indication for efficacy in Phase I safety studies
AAV-2-GAD	1E12	50	Subthalamic nucleus (STN)	GABA-mediated inhibition to affected areas	Neurologix	Modest UPDRS improvement
AAV-2-AADC	0.3E12	50-450	Putamen	Replacement missing AADC gene	Genzyme/Voyager	Modest UPDRS improvement Increased FluoroDOPA uptake
AAV-NTN	0.5E12	50	Putamen	Trophic support	Ceregene	No improvement UPDRS
AAV-NTN	2.4E12	50 + 10	Putamen + Substantia Nigra	Trophic support	Ceregene	Modest UPDRS, especially in early diagnosed patients
LV-TH-GCH-AADC	1E8 TU^∗^	50	Putamen	Internal dopamine synthesis	Oxford Biomedica	Modest UPDRS improvement
AAV-2-GDNF	3E12	450	Putamen	Trophic support	UCSF/NIH	Not yet disclosed

*^∗^Titer expressed in transducing units is different and cannot be compared to that of genomic copies of AAV.*

Moreover, alternative delivery strategies can also be undertaken using enhanced retrograde characteristics of certain vectors. For instance, the Kobayashi laboratory has developed an alternatively pseudotyped lentiviral vector that is highly efficient in transducing neurons in a retrograde manner (Hirano et al., 2013; Kato et al., 2014). By a change in the envelope of the recombinant virus, the directional transduction is evolved into a retrograde manner. This can have several implications for reaching certain areas of the brain, but also for a possible treatment of for instance motor neuron diseases (Hirano et al., 2013). These studies are very promising for future clinical application.

**FIGURE 2:**
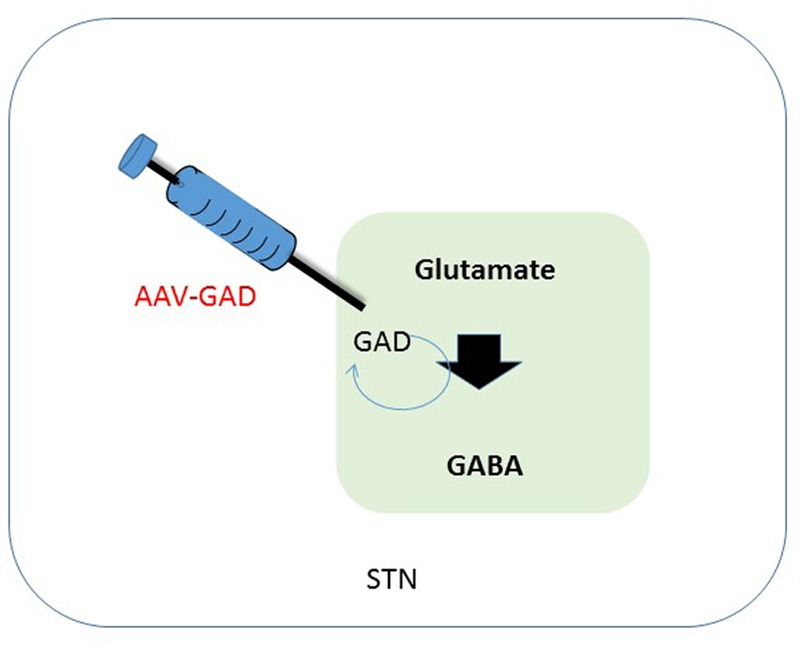
**Schematic overview of therapeutic intervention in the subthalamic nucleus.** One of the effects of a depletion of dopamine is a decreased activation of GABAergic neurons in the STN, that in their turn have an inhibitory effect. Synthesis of GABA is enzymatically done by GAD, using glutamate as a substrate. Transduction of the STN to produce GAD should lead to a restoration of GABA levels in the diseased brain, leading to less imbalance in the GABA circuitry. GABA, gama-aminobutyric acid; STN, subthalamic nucleus; GAD, glutamate decarboxylase.

An alternative target pathway for PD, the GABAergic (gama-aminobutyric acid) pathway, which is disrupted in PD, can be used. This pathway is especially affected in the subthalamic nucleus (STN). The depletion of DA and associated disturbances in GABA circuitry also affect the thalamus, a center for motor and cognitive control. Inhibition of the GABAergic pathway leads to overstimulation of thalamic pathways, a process that contributes to typical PD symptoms. The STN has served as a target for GT under the assumption that this would permit a GT approach to influence the GABAergic pathways (see **Figure 2**) (Kaplitt et al., 2007).

Several approaches have progressed to be tried in the clinic. Results are available from attempts to deliver GAD to the STN using AAV aiming to restore GABAergic balance in that area. Another approach is aimed at supplying trophic support using Neurteurin as a therapeutic molecule. For this, AAV-mediated delivery to the putamen or both putamen and directly into SN was investigated. A third approach is aiming at restoring DA synthesis by supplying DNA encoding proteins that are involved in the cascade of DA synthesis. Both lentival-based and AAV-derived vector delivery systems have been used in this approach. The lentiviral delivery approach has been used for three proteins: aromatic L-amino acid decarboxylase (AADC), tyrosine hydroxylase (TH), and GTP-cyclohydrolase I (GCH). The AAV-based approach is focusing on the delivery of AADC to compensate for the downregulation of this key enzyme in PD.

## Clinical Studies

### AAV-2-GAD

Parkinson’s disease is mostly associated with degeneration loss of dopaminergic neurons in the SN, but also of many brainstem, limbic, and midbrain neurons, which leads to alterations in the activity of brain networks that control movement. Alterations leading to dysregulation of interacting inhibitory and excitatory pathways result in a movement disorder that is characterized by difficulty initiating movements, muscular rigidity, and tremor (Lang and Lozano, 1998; Nutt and Wooten, 2005). Pharmacological facilitation of dopaminergic neurotransmission benefits most patients initially, but those with advanced PD often develop drug-related complications, such as dyskinesia and motor fluctuations. Following start of these complications, direct interventions increasing dopaminergic neurotransmission could be causing adverse effects and even worsen dyskinesia. Hence, Kaplitt et al. (2007) explored strategies that are not based on dopamine and therefore might provide benefit without these side-effects. In patients with PD, increased activity of the STN is mainly caused by a reduction of GABAergic input from the globus pallidus (Erlander et al., 1991; Obeso et al., 2000; Wichmann and DeLong, 2003). Clinical studies have shown that electrical stimulation, lesioning of the STN or infusion of GABA resulted in a reduction of STN activity. This in its turn could ameliorate signs of advanced PD (Hamani et al., 2004). GABA is the major inhibitory neurotransmitter in the brain. Glutamic acid decarboxylase (*GAD*) is one of the key enzymes catalyzing synthesis of GABA. Therefore, overexpression of GAD should lead to a decreased activity of the STN and hence less innervation of the motor circuitry (see **Figure 2**). Studies in animal models indicate that AAV-GAD improves brain function without adverse toxic events (Emborg et al., 2007).

In an open label, non-randomized phase I study, Kaplitt et al. (2007) administered an AAV-2-GAD construct via stereotactic surgery without any severe side effects. In the first series of trials, the vector was delivered unilaterally; in the following trials this was done bilaterally with a total volume of 50 μl containing 1E12 genomic copies (GC) in total (see **Table 1**). Their results show that “AAV-mediated gene transfer can be done safely in the human brain, with no evidence of substantial toxic effects or adverse events in the perioperative period and for at least 1 year after treatment. Most patients have been followed up for more than 2 years after surgery, with some for more than 3 years. No deaths and no evidence of substantial adverse events were reported” (Kaplitt et al., 2007).

This phase I study was not designed to assess the effectiveness of the intervention. Nonetheless, the authors reported encouraging clinical outcomes as determined by an increase of 3.5 points in the Unified Parkinson’s Disease Rating Scale (UPDRS). Substantial improvements in both the off and on states were evident, beginning at 3 months after surgery and continuing until the end of the trial. This improvement was localized predominantly to the side of the body contralateral to the treatment (Kaplitt et al., 2007). As most of the nerve thalamocortical fibers decussate, these results are most likely mediated by the treatment. Although an advantage of this approach is that it is dopamine independent, degeneration of dopaminergic neurons still progresses resulting in an absence of dopaminergic circuitry.

### Prosavin (LV-GCH-TH-AADC)

As an alternative to restoring the GABA balance, another approach is expressing enzymes that improve the production of DA itself. Presently two strategies are followed to achieve an improved DA production. In one approach, the key enzyme for this pathway, AADC, which is downregulated in PD patients, is supplemented via AAV-mediated GT (see **Figure 1**) (Christine et al., 2009; Mittermeyer et al., 2012). In the second approach, the three enzymes that are involved in dopamine synthesis, including AADC as well as TH and GCH, are delivered by a lentivirus-based vector (Palfi et al., 2014) (see **Figure 1**).

To overcome the loss of DA-producing neurons, an alternative route of dopamine production was developed and investigated by Palfi et al. (2014). Synthesis of DA by a source unrelated to the degenerating DA cells is feasible when the enzymes that are needed are supplemented, e.g., via a lentiviral vector. This approach does not depend on the survival of dopaminergic neurons, but uses remaining striatal neurons to produce dopamine. Retrograde transduction of diseased SN neurons projecting to the striatum, known as the nigrostriatal pathway, might also occur. Therefore, Palfi et al. (2014) developed a lentiviral approach, also known as Prosavin, carrying the three protein-encoding sequences for TH, GCH, and AADC. The rate limiting enzyme in this cascade is TH, which uses endogenous sources for synthesis of L-dihydroxyphenylalanine, also called levodopa. GCH is required as cofactor for TH. Finally, AADC converts levodopa to dopamine (Shen et al., 2000; Hadjiconstantinou and Neff, 2008; Daubner et al., 2011).

Based on this background, a clinical study was initiated in which PD patients received bilateral injections of Prosavin into the putamen. Patients were dosed in three cohorts, receiving a low [1.9E7 transducing units (TU)], mid (4E7 TU) or a high dose (1E8 TU) (see **Table 1**). Palfi et al. (2014) described an encouraging safety profile with no severe adverse events. Beneficial functional data were reported at the same time as well. In all patients, an improvement in motor score was observed. More specific, a significant improvement in mean UPDRS part III motor scores off medication was recorded in all patients at 6 months [mean score 38 (SD 9) vs. 26 (8), *n* = 15, *p* = 0.0001] and 12 months [38 vs. 27 (8); *n* = 15, *p* = 0.0001] compared with baseline. These data were in line with preclinical results (Azzouz et al., 2002; Jarraya et al., 2009; Palfi et al., 2014). No patients developed off-medication dyskinesia, whereas on-medication dyskinesia were common and were reversed by reduction in intake of oral dopaminergic therapies. This pattern is as expected for delivery of an efficacious dopaminergic therapy (Marks et al., 2008). The increased occurrence of on-medication dyskinesia in the highest dose group might correlate with the fact that this group had the highest dopaminergic delivery to the striatum, as noted in nonhuman primate models of PD (Jarraya et al., 2009). Furthermore, some of the properties of DA neurons with respect to storage, release and re-uptake of DA are lacking in non DA neurons. This might help explain the adverse neurological events and the interpretation of the changes in functional imaging.

In a long term follow-up of this study the predicted disease progression, as illustrated by a yearly decline in UPDRS part III score (off medication) of 3–4 points (Olanow et al., 2003; Eggers et al., 2012), was absent. Even though these data are very encouraging, they also fall within the range of placebo controls and hence these results should be taken with caution.

However, these data provide preliminary evidence for the safety and potential benefit for the use of lentiviral vector-mediated delivery of key enzymes for DA synthesis. One conclusion of the study investigators was that they were awaiting an improvement for the delivery and use of this vector in PD patients before they would proceed. An improvement in the delivery method could be permit an increase in delivered volume. Moreover, an improvement within the expression cassette such as codon optimization or promoter design might also be useful to increase transgene expression.

### AAV-2-AADC

With the progression of the disease status of PD, one of the main problems is that the only drug available, Levodopa (L-Dopa), becomes less effective. It has been observed that the effectiveness of L-Dopa decreases over time as levels of AADC decline (Ichinose et al., 1994; Nagatsu and Sawada, 2007). This has led to efforts to increase AADC expression using AAV-mediated gene therapy. Delivering AADC to the brain could restore the therapeutic effectiveness of levodopa and improve dopamine function.

In one study, patients received an AAV vector encoding AADC, delivered to the putamen using MRI-guided convection-enhanced diffusion (CED) delivery (Christine et al., 2009). Patients also underwent positron emission tomography (PET) scans with the AADC-specific tracer [18F]fluoro-L-mtyrosine (FMT) 1–10 days before surgery, at 1 and 6 months after surgery, and annually for up to 5 years. During the first 6 months, PET imaging revealed a significant elevation in AADC expression. This result was coupled with a good safety profile and preliminary indications of clinical benefit (Christine et al., 2009). Long term follow up of these patients demonstrated that this increase of AADC was maintained over the study period, suggesting a permanent elevation of the expression (Mittermeyer et al., 2012). This clinical study was not designed to monitor efficacy, however, temporal analyses of the UPDRS scores in the ON and OFF states showed a significant improvement in the first 12 months in all patients. This was followed by a slow deterioration over the following years. Unfortunately, no significant differences between the high- and low-dose patients were reported in either the ON or OFF score. The UPDRS showed improvement within the first 12 months. The authors indicated that the effect on the UPDRS was most likely due, at least partially, to a placebo effect (Mittermeyer et al., 2012). The placebo effect has been well documented for PD (de la Fuente-Fernandez and Stoessl, 2002). The authors are currently following up with a phase 1 study aiming for 60% transduction coverage of the putamen by increasing the volume and the dose. (ClinicalTrials.gov Identifier: NCT01973543^1^).

In parallel, another study also investigated the use of AAV-AADC for the treatment of PD. Muramatsu et al. (2010) reported an evaluation of the safety, tolerability and potential efficacy of AAV vector-mediated gene delivery of AADC into the putamen of six PD patients. Most important measures were UPDRS, and PET using a tracer for AADC. Motor score was improved with 46% in the OFF state at 6 months post-intervention, whereas PET levels increased with more than 50% and remained high up to 96 weeks, indicative of a permanent transduction of the putamen to express AADC. They stated in their report that these results warrant further evaluation in a randomized phase II study.

### Neurotrophic Factors

During development, naturally occurring neurotrophins are responsible and essential for neuronal growth, differentiation, vitality, and survival. Among the members of the neurotrophic growth factors are GDNF and NTN. Both GDNF and NTN are members of the transforming growth factor β superfamily (TGF β). They act through binding of GFRa1 or GFRa2 and subsequently signal through this GFRα-ligand complex. Together with the tyrosine kinase receptor (cRET) a functional receptor is formed that activates downstream signal transduction pathways (Jing et al., 1996; Cik et al., 2000). Activation of those pathways depends on the availability of receptors in a given brain structure and thus the biological activity depends on the availability of the receptors. During adulthood, they retain some of the vitality and survival functions that are shown in development. Both NTN and GDNF are naturally occurring proteins. They have potent neurotrophic effects on midbrain dopamine neurons, including those originating in the SN (Lin et al., 1993; Choi-Lundberg et al., 1998; Kirik et al., 2000). The possibility that these neurotrophic factors could be used to slow down, halt, or even reverse the degeneration of the DA neurons has been discussed for a long time (Lapchak et al., 1996; Björklund et al., 1997; Olanow et al., 2015a,b). This has, for instance, been shown in many animal models including lesioned nonhuman primates (Kordower et al., 2000, 2006) using neurteurin as neurotrophic factor. Beneficial effects using GDNF as a possibly therapeutic transgene have been shown in many animal models as well and moreover also been shown in aged nonhuman primates (Johnston et al., 2009).

### AAV-2-Neurteurin

CERE-120 (AAV-2-NTN) was developed with the goal of restoring function and protecting neurons from further degeneration. An initial study was performed infusing only the putamen bilaterally; a second strategy involved also infusion of the SN directly (see **Figure 1**). Results from the first phase I clinical study suggest that CERE-120 can be delivered safely to the putamen of PD patients. The safety of the stereotactical procedure was similar to that of deep brain stimulation (for review see Health Quality Ontario, 2005). In a placebo-controlled study, AAV-treated patients improved similarly to those of the control treated group, suggesting a strong placebo effect (de la Fuente-Fernandez and Stoessl, 2002). The administration of the AAV vector and its subsequent expression of the transgene did not result in any severe adverse events related to the treatment. The investigators suggested, however, that transduction profiles would improve when the vector was administered to the SN. Both strategies of infusion, putamen or putamen + SN, were followed by a phase II study. Improvements in the UPDRS were observed, similar to those of the placebo control group. Unfortunately, the results from the efficacy data for both trials were mixed and disappointing in that neither met the primary endpoint (UPDRS motor-off) within the given timeframe. A positive and surprising twist was noted by the improvement at the time points beyond the prescribed timeframe (15–18 months vs. the prescribed 12 months). This is at least an indication that time is needed to realize a treatment effect. Moreover, exploratory analysis of the data suggested that earlier stage patients (i.e., within 5 years of diagnosis) responded much better clinically to the gene therapeutic treatment than those treated after 10 or more years post-diagnosis (Bartus, 2013). This is not surprising for a rescue therapy approach; it is logical that the earlier the treatment starts, the more neurons are likely to be saved. However, the question of how quickly and when degenerating neurons are beyond rescue in an irreversibly degenerated state remains to be answered.

## Conclusion and Discussion

Successful delivery of the transgene is a key consideration for all of the above-described studies to date. So far, only AAV-2 or lentiviral vectors have been used. Both vector systems are capable of transducing neurons in the CNS. The delivery itself is highly dependent on the target structure.

The STN is compared to the putamen rather small and thus requires less vector. In the putamen, several different volumes have been infused, ranging from 50 to 450 μl per side. The lowest amount of vector was used as starting point, and from there volumes have increased. Also the addition of a CED delivery method has increased the spread of vector. Improvements in catheter design, using a so-called step catheter to prevent backflow have added to the improvement of vector delivery. For a full coverage of the structure, these parameters have to be taken into account for a successful delivery. Finally, with the aid of imaging techniques, the injection can be followed under MRI-guidance. This has a significant impact on the safety of the procedure as certain structures can be avoided during the surgery and the precision of the placement of the needle can be better controlled (Richardson et al., 2011, for review, see Salegio et al., 2012).

One of the merits of using growth factors, such as NTN or GDNF is that the disease etiology may not be relevant. Halting the process of degeneration is not per definition reliant on the cause of disease. However, one needs to keep in mind that the accumulation of for instance molecules such as alpha-synuclein may cause the transport mechanisms to stall, hindering delivery of trophic factors in a rodent model of alpha-synuclein overexpression (Decressac et al., 2011). More sophisticated studies remain to be undertaken to fully understand the transport mechanisms in a PD diseased brain. For both GAD and AADC-mediated therapies, the therapy is not related to the number of surviving neurons, so patients with more advanced disease may be helped with this type of therapy. Moreover, in contrast to the use of GAD or Prosavin, neurotrophin therapy is a so-called disease modifying as the aim of the therapy is to interfere in the degeneration process. Neurons that are still present are helped with a better neurotrophic factor balance. On the other hand, when all remaining neurons have been affected, the other options may be the therapy of choice. In general, for a neuroprotective therapy, one needs the maximal number of remaining neurons.

The strategy that comprises the use of GAD is a form of treatment aiming at a more physiological method to set basal ganglia motor circuitry to return to a normal state, as shown in preclinical studies (Luo et al., 2002). Indeed, using deep brain stimulation of the STN some improvement in the ON state UPDRS is observed, however, the effect of the gene therapy is much higher (Kleiner-Fisman et al., 2006). This observation lends support to this possibility of reinstating homeostatic physiological control.

Results from the clinical trials may at a first look seem somewhat disappointing, but have brought the field to a state that safe delivery of viral vectors into the brain is feasible. Expression of a foreign (therapeutic) gene is possible and in none of the studies, long term exposure to the transgene has led to severe adverse events. With such a progression, delivery of the silver bullet still seems within reach.

## Author Contributions

BB and HP wrote the manuscript.

## Conflict of Interest Statement

The authors declare that the research was conducted in the absence of any commercial or financial relationships that could be construed as a potential conflict of interest.

## Footnotes

1 https://clinicaltrials.gov/ct2/show/NCT01973543
